# Machine Learning-Assisted Network Inference Approach to Identify a New Class of Genes that Coordinate the Functionality of Cancer Networks

**DOI:** 10.1038/s41598-017-07481-5

**Published:** 2017-08-01

**Authors:** Mehrab Ghanat Bari, Choong Yong Ung, Cheng Zhang, Shizhen Zhu, Hu Li

**Affiliations:** 1 Department of Molecular Pharmacology and Experimental Therapeutics, Mayo Clinic College of Medicine, Rochester, MN 55905 USA; 2 Department of Biochemistry and Molecular Biology, Mayo Clinic College of Medicine, Rochester, MN 55905 USA

## Abstract

Emerging evidence indicates the existence of a new class of cancer genes that act as “signal linkers” coordinating oncogenic signals between mutated and differentially expressed genes. While frequently mutated oncogenes and differentially expressed genes, which we term Class I cancer genes, are readily detected by most analytical tools, the new class of cancer-related genes, i.e., Class II, escape detection because they are neither mutated nor differentially expressed. Given this hypothesis, we developed a Machine Learning-Assisted Network Inference (MALANI) algorithm, which assesses all genes regardless of expression or mutational status in the context of cancer etiology. We used 8807 expression arrays, corresponding to 9 cancer types, to build more than 2 × 10^8^ Support Vector Machine (SVM) models for reconstructing a cancer network. We found that ~3% of ~19,000 not differentially expressed genes are Class II cancer gene candidates. Some Class II genes that we found, such as SLC19A1 and ATAD3B, have been recently reported to associate with cancer outcomes. To our knowledge, this is the first study that utilizes both machine learning and network biology approaches to uncover Class II cancer genes in coordinating functionality in cancer networks and will illuminate our understanding of how genes are modulated in a tissue-specific network contribute to tumorigenesis and therapy development.

## Introduction

Although cancers arise as uncontrolled clonal expansion of affected cells^1, 2^, they are increasingly acknowledged as diseases of biological systems^3^ or diseases of cell ecology^4, 5^. Hallmarks of the combinatorial actions of genes in cancer networks and cell-cell interactions in their microenvironments include uncontrolled cell proliferation, invasiveness, deregulated cellular energetics, and altered cell-cell communications^6^. While the culprits driving such cancer hallmarks have been thought to be frequently mutated oncogenes and/or differentially expressed genes, emerging evidence indicates a new class of genes may also be playing a role.

Based on the prediction of a mathematical cancer evolutionary model, which simulates cancer growth and survival fitness, Gatenby *et al*. recently demonstrated that targeting “never mutated” genes might be effective in halting cancer progression^7^. Their mathematical simulation study suggested combination therapies sequentially targeting a never-mutated gene and then a compensating-driver-mutation can be substantially more effective than conventional treatments.

Moreover, continuous connections between differentially expressed genes and mutated oncogenes^8, 9^, which we term Class I cancer genes, have not been revealed by pan-cancer network analyses, suggesting the existence of a new class of cancer genes (Class II cancer genes) that act as “signal linkers” in coordinating oncogenic signals between mutated and differentially expressed genes. These Class II cancer genes, which have escaped detection by conventional methods because they are neither differentially expressed nor mutated (or rarely mutated), act as invisible hands in “connecting the dots”^10^ to complete oncogenic signals in a cancer network. Of the approximately 20,000–25,000 genes in the human genome^11, 12^, we hypothesize Class II cancer genes may represent only a small portion in human genome because most non-differentially expressed or non-mutated genes are “neutral” in tumorigenesis.

We therefore seek to employ machine learning methods with proven power to extract useful information from multi-dimensional data at high-dimensional spaces^13^ to identify cancer-related Class II gene candidates. Machine learning approaches have been broadly applied to classify biological samples^14^, characterize pharmacological agents^15, 16^, and identify functional genetic elements in gene regulation^17–19^. However, to our knowledge, the strengths of both machine learning and network biology approaches have yet to be integrated in an attempt to show how a repertoire of cancer genes coordinate to give rise to cancerous properties.

In this work, we aim to use a hybrid approach that harnesses the power of both machine learning and network biology to provide new insights and improve understanding of cancer etiology, particularly related to the existence of Class II cancer genes. To test our hypothesis and approach, we used transcriptomics data derived from 9 different cancer types (breast, colon, kidney, liver, lung, ovarian, pancreatic, prostate, and skin). Our network analyses revealed the existence of Class II cancer gene candidates residing in networks reconstructed from these cancer types. Further reconstruction of genetic landscapes for ovarian, breast, and pancreatic cancers revealed that many previously reported mutated oncogenes are indeed surrounded by Class II cancer gene candidates where they play a role as “mediators” to convey oncogenic signals in cancer networks. Taken together, we provide the first delineation of cancer networks using a machine learning approach, and we uncover the potential roles of Class II cancer genes in cancer etiology. We found this new kind of machine learning-based reverse engineered cancer networks can be complemented with networks derived from protein-protein interactions, correlation-based reverse engineered networks, and cross-omic networks using tool such as KeyPathwayMiner 4.0^20^ as well as other de novo pathway enrichment methods, with guidelines provided by Batra *et al*.^21^ for network-based novel discoveries.

## Results

### Reverse engineering high-dimensional cancer networks via a machine learning-based network inference approach

**Figure 1:**
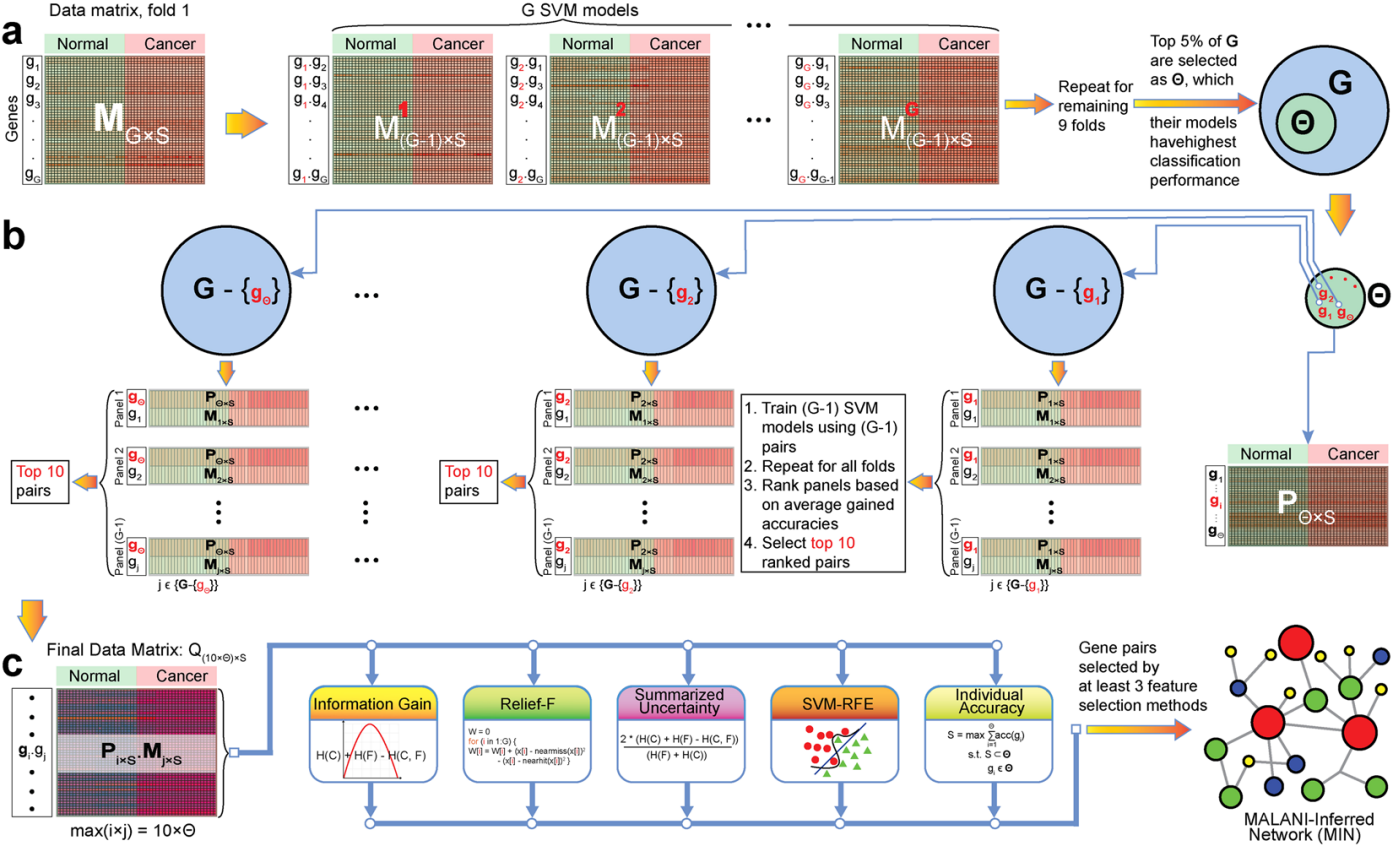
Overview of Machine Learning-Assisted Network Inference (MALANI) algorithm to infer a cancer network for a given cancer type. The overall MALANI procedure consists of three major stages: (**a**) Stage 1: Construction of gene-wise models. Step 1.1: Split gene expression data matrix for 10-fold cross-validation. Original gene expression matrix is represented as ***M***_*G*×*S*_ where *G* represents the number of genes (rows in data matrix) and *S* represents samples (column in data matrix). The gene expression matrix was split into 10 portions, each for cancer and normal samples. Step 1.2: Construct gene-wise dot product matrices. For the first training fold, gene-wise dot product matrices are constructed from split data from step 1.1. Step 1.3: Model training and testing. Gene-wise matrices generated from Step 1.2 were used for training and the remaining one split portion as testing. The step was then repeated 9 times for 10-fold cross-validation (see Materials and Methods for more detailed description). Step 1.4: Evaluation of gene-wise model performance. Performance of gene-wise dot product models were assessed for their capability to classify cancer vs. normal samples. The top 5% gene-wise models with the best classification performance that constitute a gene set of **Θ** were selected. (**b**) Stage 2: Construction of gene-pair models. Step 2.1: Construction of gene-pair vector from **Θ** set. Using data from Step 1.1, vectors of gene pairs from a set of **Θ** genes obtained from Step 1.4 (denoted as red “g”) with remaining genes in the expression array (denoted as black “g”) were constructed. Step 2.2: Model training and testing. Each vector corresponding to a gene in set **Θ** were used to train SVM models for each training fold and 10-fold cross-validation procedure as described in Step 1.3 was performed. Step 2.3: Evaluation of gene-pair model performances. Gene-pair model performance was assessed based on model’s capability to classify cancer vs. normal samples. Genes not found in the **Θ** set (black “g”) that paired with genes in the **Θ** set (red “g”) and were among the top 10 in terms of best classification performance were deemed functionally associated with a given gene in set **Θ**. (**c**) Stage 3: Inferring cancer networks. Step 3.1: Construction of finalized gene-pair dot product matrix. Step 3.2: Assessment of gene pair performance in classifying cancer samples. Five feature selection methods were used to assess performance of each gene pair dot product in classifying cancer vs. normal samples. Step 3.3: Construction of MALANI-Inferred Network (MIN) for a cancer. Selected gene pairs by at least three different feature selection methods in Step 3.2 were then agglomerated to reconstruct an inferred cancer network.

To provide mechanistic insights into cancer etiology, we developed a Machine Learning-Assisted Network Inference (MALANI) method to identify cancer-associated gene pairs, which are the foundation of cancer network construction. Unlike most conventional network inference methods^8, 22, 23^, where downstream analyses rely on finding the functional associations among genes or biological pathways with differentially expressed or mutated genes, MALANI makes no assumption that differentially expressed or mutated genes distinguish the biology of cancers from normal tissues. Instead, MALANI transforms dot products of expression values of gene pairs into high-dimensional spaces using a machine learning approach termed Support Vector Machine (SVM)^24^ (Materials and Methods). The advantage of this strategy is that all discoveries made are unbiased in the sense that the role of all genes (not merely differentially expressed and mutated genes) are equally assessed in the context of cancer etiology, thus facilitating discovery of yet-to-be-identified cancer-causing genes. An overview of how the MALANI algorithm can be used to infer a cancer network is provided in Fig. 1. While SVM is the choice of machine learning method used in this work, we also demonstrated that the MALANI algorithm is equally applicable to other machine learning and feature selection approaches (Materials & Methods).

### Cross-cancer network analysis reveals tumor-associated and cancer type-specific molecular properties

**Figure 2:**
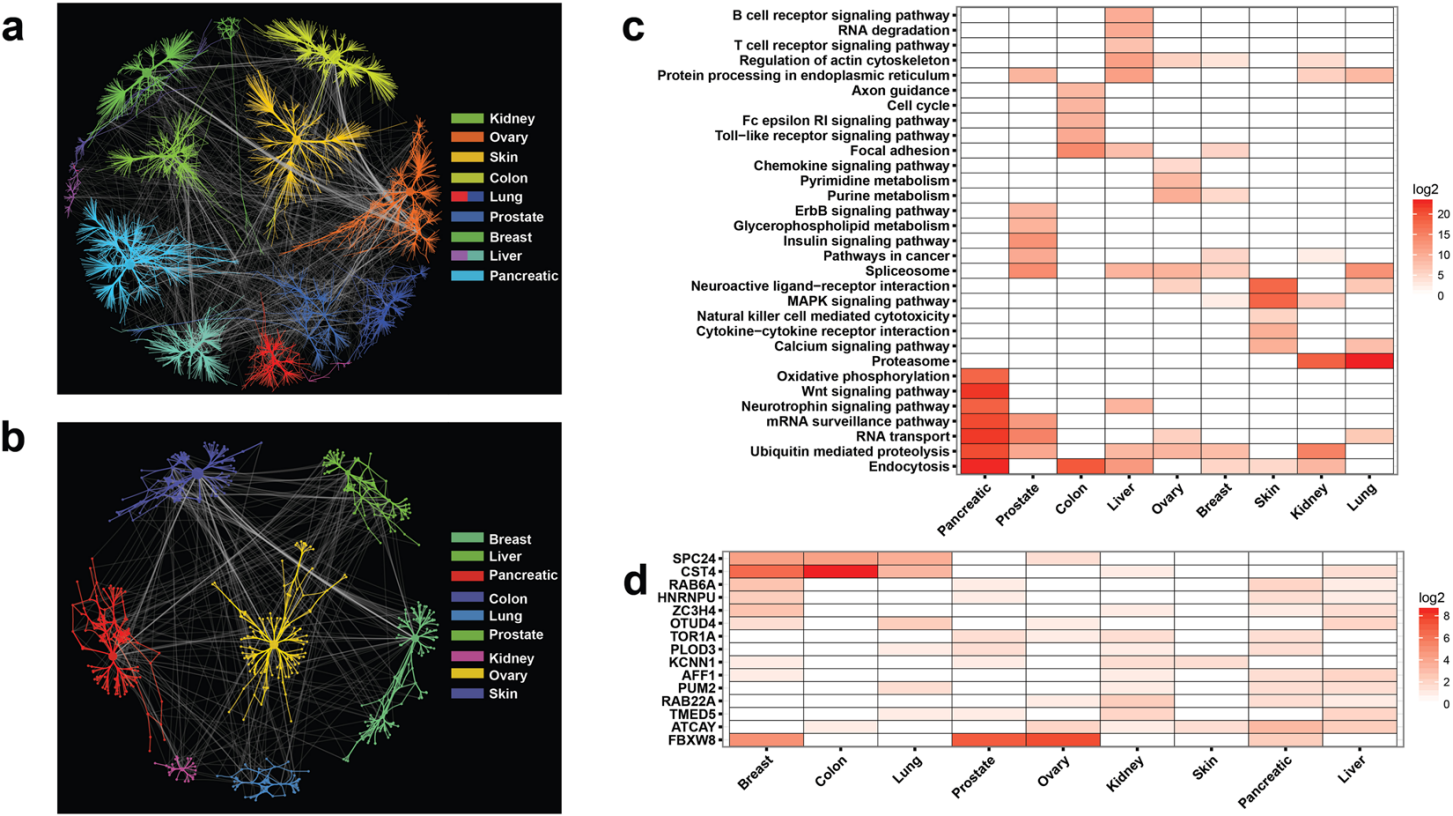
The properties of MALANI-Inferred Networks (MINs) across 9 cancer types. (**a**) Complete MINs reconstructed across cancer types via agglomeration of gene pairs selected by at least three different feature selection methods. Color codes denote different cancer types. (**b**) Shared gene pairs found across at least two cancer types indicated. (**c**) Top 10 enriched canonical pathways for genes residing in MIN for each cancer type. Heatmap plot based on negative of enrichment p-values at log2 scale. (**d**) Heatmap for genes found in MINs of at least 4 different cancer types. Heatmap plot based on the log2 scale of frequency, i.e. number of a given gene appearing in different gene pairs of an inferred network.

Here we asked the effect of tissue-specific context to the model performance in classifying cancer from different tissue types by using an unified normal samples (i.e. normalized across all tissue types). We evaluated our model performance using normalized normal samples across pancreas, prostate, and liver tissues as single unified normalized normal tissues for their capability in classifying pancreatic, prostate, and liver cancers. In general, our results indicate a drop of classification performance when unified normal samples were used (see Supplementary Discussion for further description). To investigate whether gene pairs within the MALANI-inferred networks (MINs) are enriched in known cancer-related pathways and across cancer types, we constructed an inferred network that unifies the respective networks of 9 cancer types (See Supplementary Table 1 and Supplementary Data 1). **G**_**i**_-**G**_**j**_ gene pairs selected by at least three feature selection methods at Stage 3 of the MALANI algorithm (Fig. 1c) were used as input (Fig. 2a, gene pairs for each cancer type are provided in Supplementary Data 2). Of 3377 unique genes identified in the networks (Supplementary Figure 1), 579 appeared in at least two cancer types (Fig. 2b). Most of these gene pairs (or edges) in the unified, inferred network are cancer-type specific (Fig. 2a and see also Supplementary Discussion). Our finding is consistent with the fact that tissue-specific contexts are important in cancer etiology^25^.

We next performed pathway enrichment analyses to evaluate cancer-related properties of our network models. Our pathway enrichment analyses (Fig. 2c) revealed the following pathways were enriched in at least three cancer types: (i) protein homeostasis (protein processing in endoplasmic reticulum, ubiquitin-mediated proteolysis), (ii) cell-cell interactions and morphology (regulation of actin cytoskeleton, focal adhesion), (iii) RNA homeostasis (spliceosome, RNA transport), (iv) cancer-related signaling pathways (pathways in cancer, MAPK signaling pathway), and (v) others (neuroactive ligand-receptor interaction, endocytosis). Processes involved in both protein and RNA homeostasis as well as endocytosis, with known roles in tumorigenesis^26–30^, were common enriched processes in at least 4 of the different cancer types examined.

We found a total of 15 genes present in the MALANI-inferred networks (MINs) of at least four cancer types (Fig. 2d). These genes fall into the following functional categories: kinectochore complex (SPC24), Ras-related oncogene (RAB6A, RAB22A), post-transcriptional RNA processing (HNRNPU, PUM2), transcriptional regulation and chromatin remodeling (ZC3H4, AFF1), ubiquitin-mediated proteolysis (OTUD4, FBXW8), chaperone and protein folding (TOR1A), cell structure integrity (CST4, PLOD3), ion conduction (KCNN1, ATCAY), and vesicular protein trafficking (TMED5). In general, our pathway enrichment analyses indicated cancer-related processes are indeed enriched in MINs.

To further assess the specificity of our models, we collected 288 liver cancer arrays and 217 ovarian cancer arrays not used in our initial model building as independent data test sets (Supplementary Data 3). In using feature genes selected at Stage 1 of the MALANI algorithm, we found there are a number of feature genes shared among different cancer types (Fig. 2b), indicating feature genes selected from one cancer type might also exhibit good classification performance in another cancer type. Our results indeed show high prediction accuracies for all samples, with selected gene features corresponding to liver and ovarian cancers among the highest performing (“Independent tests,” left-most table, Supplementary Data 3). Because the number of normal samples are limited in these independent data test sets (10 out of 182 for liver cancer; 10 out of 217 for ovarian cancer), the capability of our trained model to correctly classify normal samples is crucial in determining specificity. Our results show that selected gene features for liver and ovarian cancers are capable of correctly classifying a small number of normal samples out of large cancer samples (“Independent tests,” right-most table, in Supplementary Data 3). The fact that prediction performance to correctly classify normal samples is comparatively poor for gene features selected for other cancer types indicates selected gene features in our models indeed capture oncogenic signals relevant to tissue of cancer origin.

### MALANI-inferred networks (MINs) represent discovery platforms for Class II cancer genes

Having shown MINs are indeed relevant to cancer, we sought to determine whether Class II cancer genes may be identified in MINs. Given studies indicating known cancer-causing genes are not always overexpressed in the tissue of tumor origin^31^, other factors, specifically the cellular context, may dictate the contribution of these genes to tumorigenesis^25^. We therefore evaluated the inferred networks for each cancer type to determine the relative contributions of differentially expressed genes and Class II cancer genes, which influence cellular context. As shown in Supplementary Table 2, we found 1015 (in liver cancer) to 2987 (in ovarian cancer) of 20075 genes are up-regulated (cancer samples >1.5 fold change than normal controls) and 590 (in prostate cancer) to 3195 (in ovarian cancer) of 20075 genes are down-regulated (normal control samples >1.5 fold change than cancers). Of the differentially expressed genes, only a small proportion of up-regulated genes (26 such genes in pancreatic cancer; 138 in lung cancer) and down-regulated genes (12 genes in colon cancer to 97 genes in lung cancer) are constituents of MINs (Supplementary Table 2), indicating not all differentially expressed genes are equally important in tumorigenesis. Non-differentially expressed genes that improve classification performance of SVM models, making it possible to distinguish between cancers vs. normal samples, are potential Class II cancer gene candidates. In fact, we found such Class II cancer gene candidates constitute 0.91% (144 genes in lung cancer MIN) to 2.93% (467 genes in skin cancer MIN) of total non-differentially expressed genes in cancer-related MINs (Supplementary Table 2). There is only a small drop in the percentage of Class II genes in MINs even under the criterion of 1-fold differential expression (i.e. almost no differential expression with normal samples), indicating expression of most Class II gene candidates considered under 1.5-fold criterion are indeed close to normal samples (Supplementary Table 2). Our results show a small proportion (typically < 3%) of non-differentially expressed genes are Class II cancer gene candidates that modulate the activities of cancer networks. Thus, MALANI is capable of distinguishing cancer-related Class II gene candidates from their non-cancer-related counterparts.

### Hub genes in MINs are mainly differentially expressed Class I cancer genes acting as potential key mediators in cancer networks

Since hub genes that show high connectivity in a network play a key role in modulating network properties, we sought to determine whether hub genes residing in MINs are cancer-related. Here, we define genes exhibiting connectivity higher than 5 in our network models as hub genes. We found MIN hub genes are almost always differentially expressed genes in cancers (Supplementary Figure 1), indicating the importance of Class I cancer genes (mutated and/or differentially expressed) in cancer etiology. Given not all differentially expressed genes are equally important in cancer etiology, the MALANI algorithm approach is capable of condensing the pool of differentially expressed genes to only those of functional significance to tumorigenesis.

Further analysis of the annotated functions of these hub genes revealed the following cancer-related functional categories: (i) signaling processes that regulate cell proliferation, morphogenesis and differentiation (ZCCHC12 in pancreatic cancer, WNT2 in colon cancer, GAS6 in kidney cancer); (ii) RTK signaling (SHKBP1 that inhibits CBL-SH3KBP1 complex mediated down-regulation of EGFR signaling in prostate cancer); (iii) G-protein-coupled receptor (GPCR) signaling (LINGO1 in breast cancer, P2RY6 in lung cancer); (iv) transcriptional regulation (ZCCHC12, HOXA7 in pancreatic cancer, DMRTA1 in lung cancer); (v) ubiquitination and proteasome degradation (FBXW8 in both prostate and ovary cancers); (vi) cell adhesion (CLDN1, which plays a role in tight junction, and collagen COL11A1 in colon cancer, S1PR1 in lung cancer, STAB2 and OLFML2B in liver cancer, HRG in kidney cancer, A1BG in skin cancer); (vii) secreted factors (CST4 that acts as a proteinase inhibitor in secreted fluids in colon and breast cancers); (viii) cellular metabolism and energetic (the CYP3A7-CYP3A51P readthrough in breast cancer, MIOX in lung cancer, MRPL15 in ovary cancer); (ix) immune-related and inflammatory responses (P2RY6 in lung cancer; CLEC4M in liver cancer). Because hubs link to a number of Class II cancer gene candidates, the functionality of hubs (which are Class I cancer genes) most likely determined (or is determined by) the functionality of the associated Class II cancer genes.

### Protein-protein interactions encapsulated by gene pairs in MINs recapitulate known cancer-related biological processes and key genes in mediating cancer homeostasis

**Figure 3:**
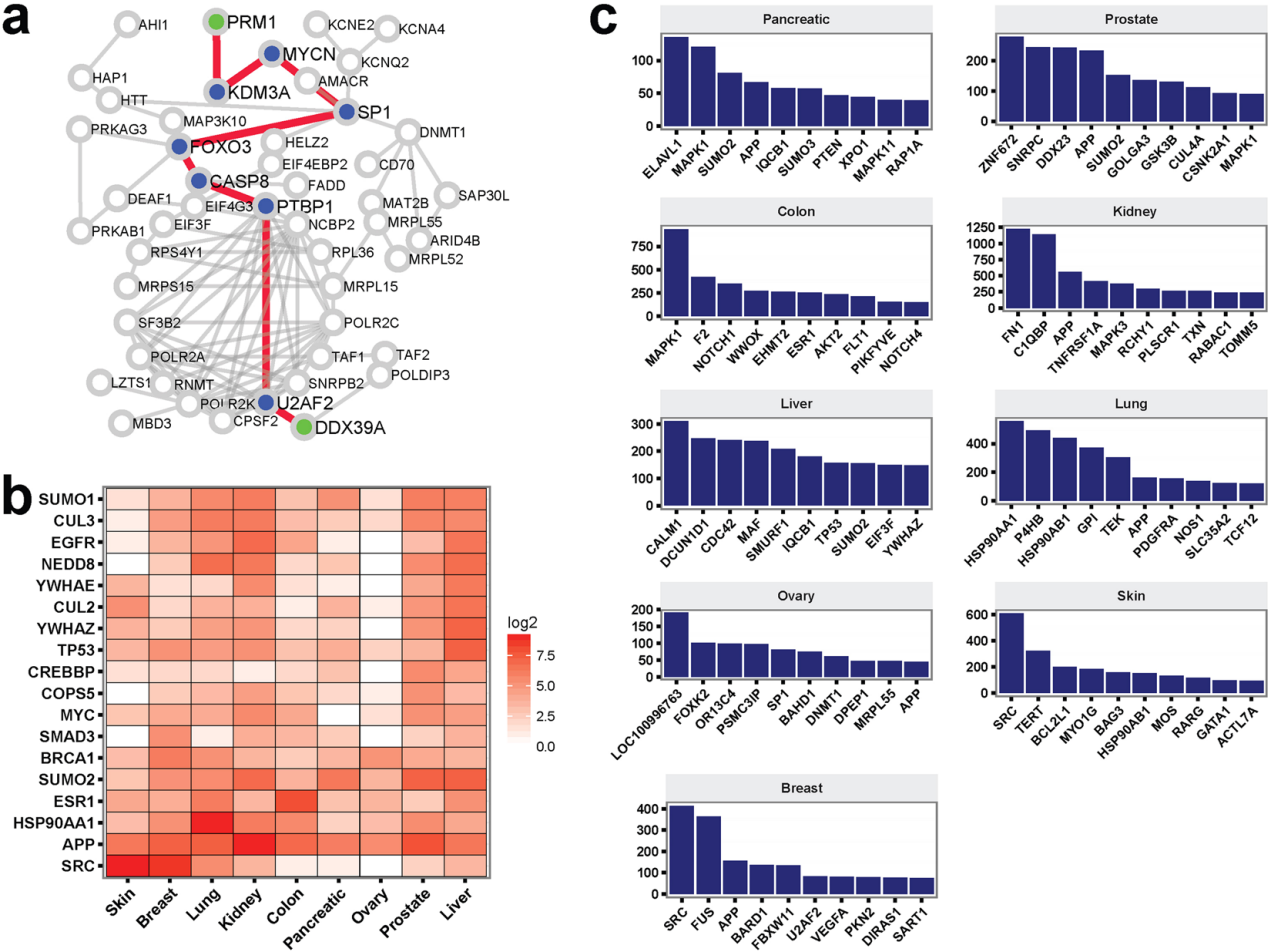
The reconstruction and properties of Protein-Protein Interactions Encapsulated by MINs (PIE-MINs) across different cancer types. (**a**) The construction of PIE-MIN. Here, the gene pair DDX39A-PRM1 (green nodes) from an ovarian cancer inferred network is used as an illustrative example. Of more than ~1,000,000 possible undirected paths connecting a given gene pair, the shortest protein-protein interaction path (red edges) is computed using Dijkstra’s algorithm. The procedure is repeated for all gene pairs in the MIN; proteins found within the shortest path (blue nodes) between all gene pairs comprise the complete PIE-MIN for a given cancer. (**b**) Genes universally observed in all 9 cancer PIE-MINs. Heatmap plot based on the frequency of the shortest paths passing through a given gene for all gene pairs in a MIN at log2 scale. (**c**) Enriched canonical pathways for PIE-MIN using genes of top 500 frequencies. Heatmap plot based on the negative of enrichment p-values at log2 scale. (**d**) PIE-MIN genes of top 10 highest frequencies that appeared in shortest paths for all gene pairs in a MIN for each cancer type.

To illuminate the mechanistic role of Class II genes in cancer etiology, we next sought to identify protein-protein interaction paths connecting MIN gene pairs; the paths can serve as important functional counterparts for MIN gene pairs, which do not necessarily directly interact. The shortest protein-protein interaction paths connecting gene pairs were deemed functionally relevant (Fig. 3a). We termed the expanded inferred network comprised of the **G**_**i**_-**G**_**j**_ gene pairs and the corresponding, shortest protein-protein interaction paths Protein-Protein Interactions Encapsulated by MIN (PIE-MIN). We found 18 genes (ubiquitin C (UBC) excluded) were universally included across all cancer-type networks (Fig. 3b). These genes play a role in the following cellular functions: (i) RTK and 14-3-3 signaling (EGFR, YWHAE, YWHAZ, SRC); (ii) transcriptional regulation (CREBBP, MYC, TP53, SMAD3, ESR1); (iii) DNA damage and repair (TP53, BRCA1); (iv) protein quality control that involves folding and degradation (SUMO1, SUMO2, CUL2, CUL3, NEDD8, COPS5, HSP90AA1, APP). The high inclusion frequency of these well-known oncogenes and genes reported to play a role in tumorigenesis in protein-protein interaction paths connecting MIN gene pairs suggest these genes, including Class II cancer gene candidates residing in MINs, indeed play a key role in cancer etiology.

Given frequently visited proteins in the shortest protein-protein interaction paths are most likely important genes in tumorigenesis, we queried the top 10 genes that exhibited the highest frequencies encountered in shortest paths connecting gene pairs in MINs (Fig. 3c). APP and SUMO2, which play an important role in protein homeostasis with reported involvement in cancer^32–34^, and MAPK1, a kinase that regulates cell cycle and is well-known for its roles in cancers, were among the top 10 genes exhibiting the highest frequencies in PIE-MINs of at least two cancer types. Other top frequency genes, though more specific to a given cancer type but nonetheless related to cancerous properties, were also uncovered (Fig. 3c). For example, we identified exportin 1 (XPO1), which mediates leucine-rich nuclear export signal (NES)-dependent protein transport, in the pancreatic cancer PIE-MIN; thioredoxin (TXN), which mediates many redox reactions in cells and is reportedly associated with a number of cancers^35, 36^, in the kidney cancer PIE-MIN; Notch homolog 2 N-terminal-like protein (LOC100996763) and Bromo Adjacent Homology Domain Containing 1 (BAHD1) and DNA methyltransferase (DNMT1), which modulates chromatin remodeling, in the ovarian cancer PIE-MIN; and squamous cell carcinoma antigen recognized by T-Cells 1 (SART1) in the breast cancer PIE-MIN.

### PIE-MIN genetic landscapes highlight key mutation types in ovarian, breast, and pancreatic cancers

**Figure 4:**
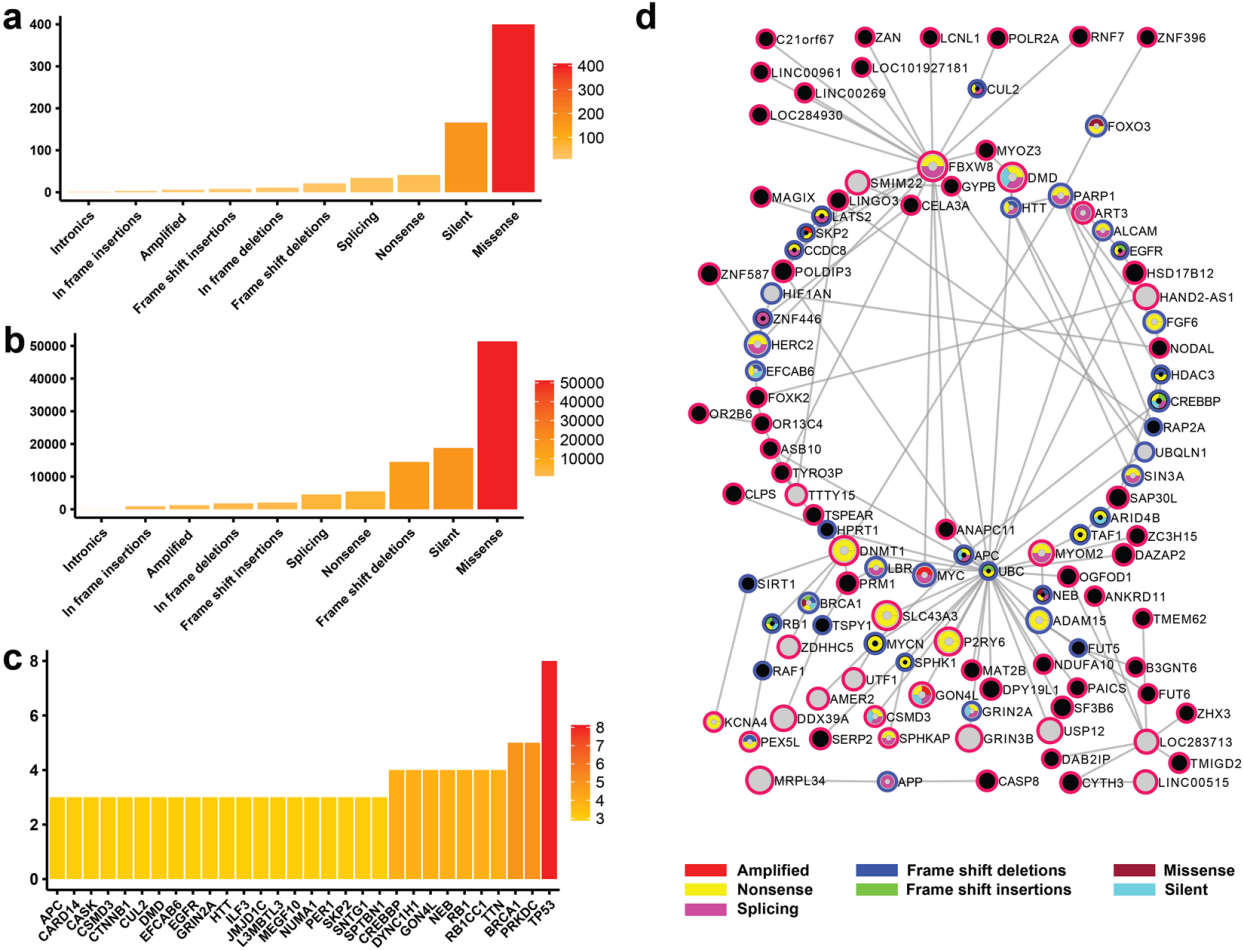
Reconstructed genetic landscape of ovarian cancer PIE-MIN. (**a**) Frequency of a given mutation type mapped to ovarian cancer PIE-MIN. (**b**) Cumulative number of protein interacting partners by mutation type in ovarian cancer PIE-MIN. (**c**) Frequency of mapped genes showing at least three different mutation types mapped to ovarian cancer PIE-MIN. (**d**) Presence of cancer-related Class I and Class II genes. Ovarian cancer PIE-MIN enriched with mutated genes from a genome-wide study implying the importance of Class I oncogenes in cancer network. Genes with detected mutations indicated by node color codes corresponding to types of mutations they display. Genes identified from protein-protein interactions within a MIN indicated by blue borders. Grey nodes with red borders indicate differentially expressed genes, which are also Class I cancer genes; black nodes indicate not differentially expressed genes; black nodes with red borders denote Class II cancer gene candidates.

**Figure 5:**
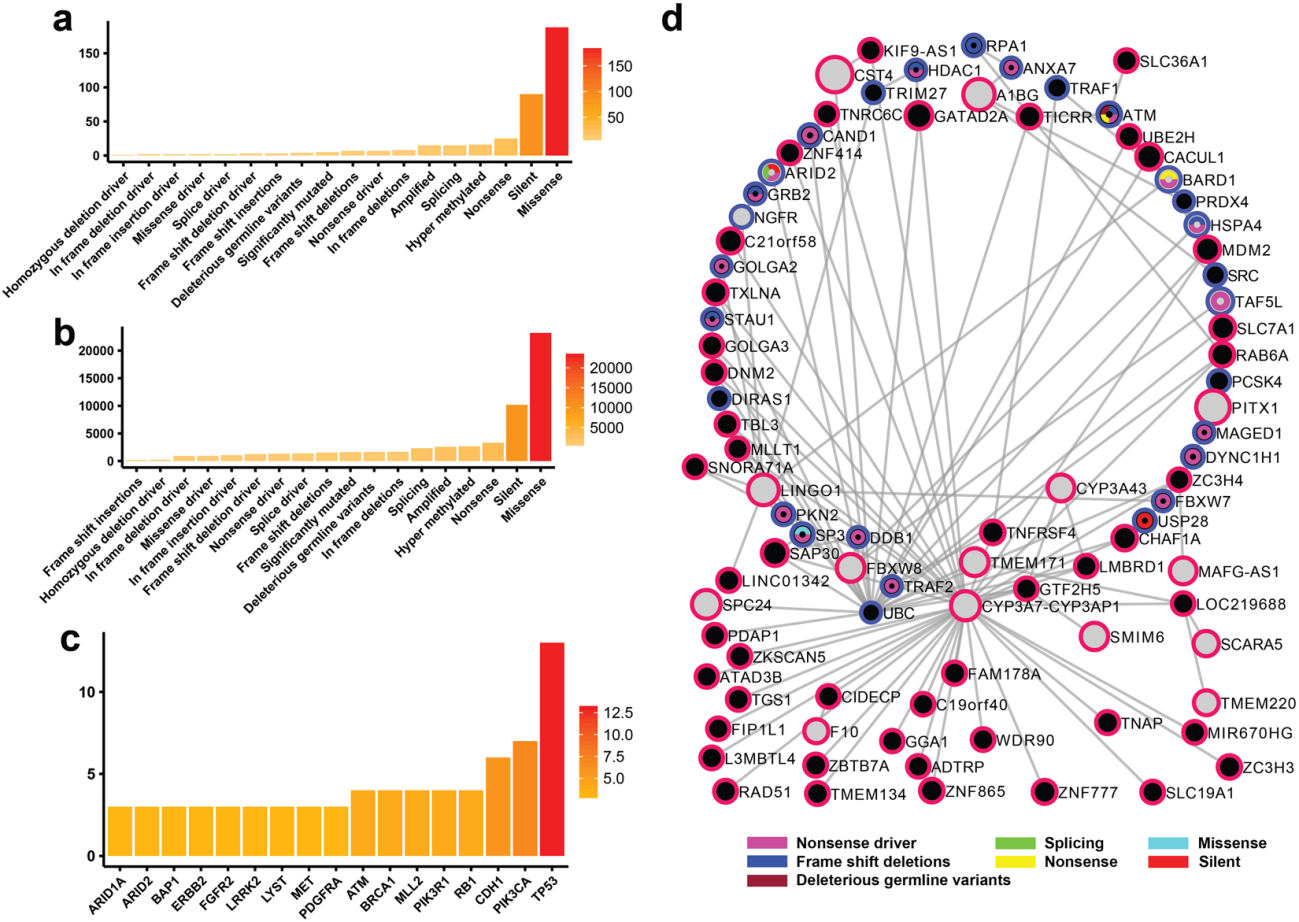
Reconstructed genetic landscape of breast cancer PIE-MIN. The reconstructed breast cancer PIE-MIN illustrates the (**a**) frequency of a given mutation type; (**b**) cumulative number of protein interacting partners by mutation type; (**c**) frequency of mapped genes showing at least three different mutation types; (**d**) Presence of cancer-related Class I and Class II genes.

**Figure 6:**
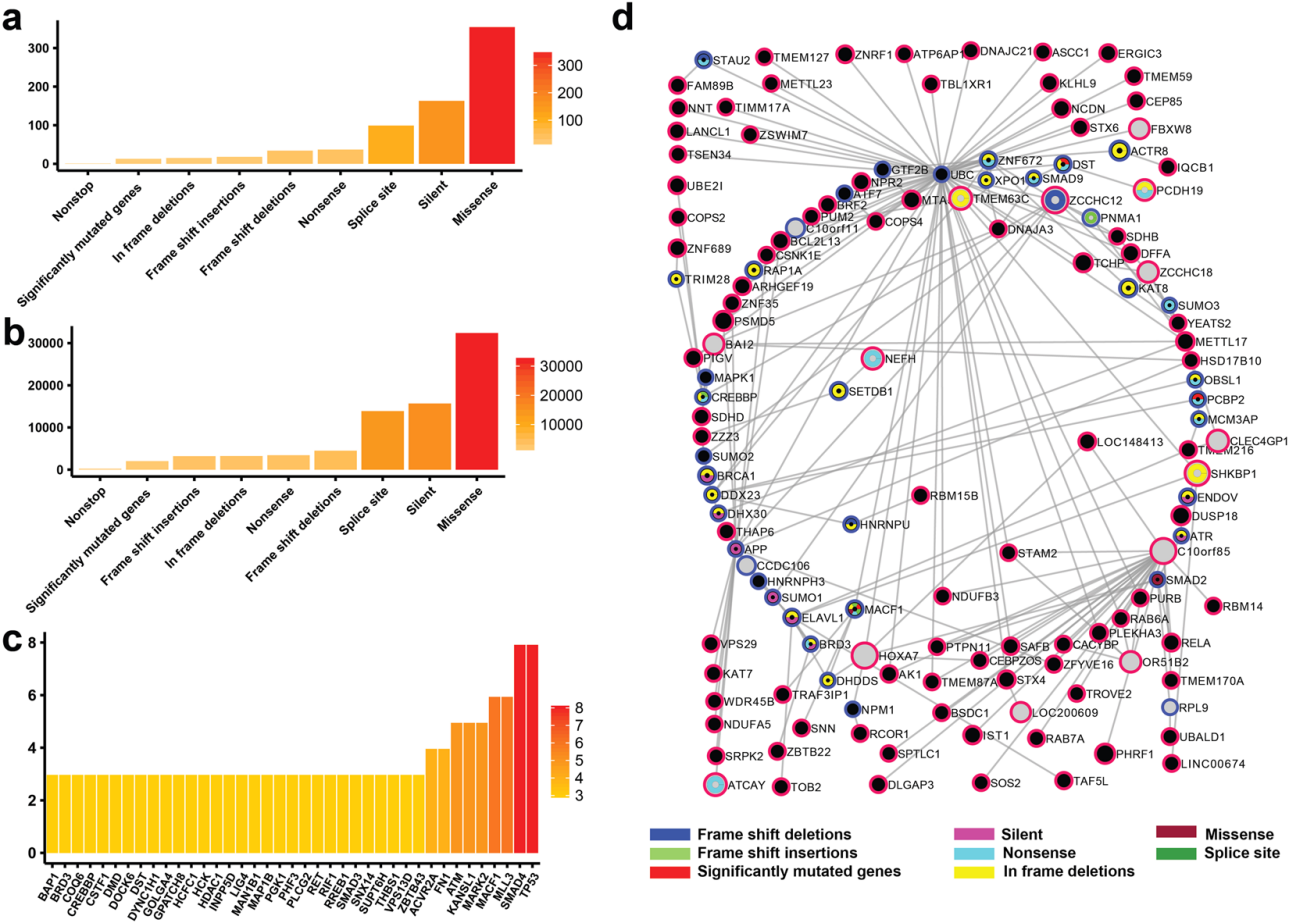
Reconstructed genetic landscape of pancreatic cancer PIE-MIN. The reconstructed pancreatic cancer PIE-MIN illustrates the (**a**) frequency of a given mutation type; (**b**) cumulative number of protein interacting partners by mutation type; (**c**) frequency of mapped genes showing at least three different mutation types; (**d**) Presence of cancer-related Class I and Class II genes.

To determine whether the networks inferred from our model reflect the reported molecular portraits of certain cancers, we evaluated whether our networks capture reported mutations from genome-wide studies in ovarian^37^, breast^38, 39^, and pancreatic^40^ cancers. We mapped known mutated genes (Supplementary Data 4) onto PIE-MINs of ovarian, breast, and pancreatic cancers, respectively (Supplementary Data 5). In order of frequency, we found missense mutations, silent mutations, nonsense mutations, and splicing mutations among the top enriched mutation types in ovarian (Fig. 4a), breast (Fig. 5a), and pancreatic (Fig. 6a) cancers. The broad coverage of mutated genes and consistency across mutational profiles indicate that PIE-MINs indeed capture essential mutation-mediated tumorigenesis mechanisms as reported by previous studies. Moreover, missense and silent mutations rank among the top 2 mutation types exhibiting the highest number of protein interacting partners in all these three cancers. Given the ranking in terms of mutation types (Figs 4a and 6a) corresponds with the number of protein interacting partners per mutation type (i.e. the greater the mutation type frequency, the greater the number of cumulative protein interacting partners) (Figs 4b and 6b), suggests their importance in a cancer network.

In identifying Class I frequently mutated genes that are key players in cancer networks, we found TP53, which is found in all inferred networks regardless of cancer type and plays a known role in tumorigenesis, exhibits more mutation types than any other gene in ovarian (Fig. 4c), breast (Fig. 5c), and pancreatic (Fig. 6c) cancers. In addition, we identified multiple types of tumor suppressor gene (RB1 and BRCA1) mutations in both ovarian (Fig. 4c) and breast (Fig. 5c) cancer networks, which is consistent with findings that mutations in RB1 and BRCA1 are extremely important in the progression of ovarian and breast cancers. PIK3CA and CDH1, which are reported to be implicated in breast cancer development^38^ ranked second and third followed by genes involved in RTK signaling, such as PDGFRA, FGFR2, ERBB2, and MET (Fig. 5c). SMAD4 and ACVR2A, which are involved in TGF-β signaling, and histone-modifiers MLL3 and HDAC1 are among the top genes showing multiple mutation types in the inferred pancreatic cancer network (Fig. 6c), which is consistent with a previous study^40^.

### Class II cancer genes are functional coordinators between Class I differentially expressed hubs and highly mutated genes

Having reconstructed mutational genetic landscapes for PIE-MINs of ovarian, breast, and pancreatic cancers, we next asked whether there are Class II cancer genes – those that are neither differentially expressed nor mutated – present and playing a role in connecting Class I differentially expressed hubs and frequently mutated genes. Our statistical analysis (Supplementary Table 3) showed there are indeed a substantial number of Class II genes (denoted by black nodes with a red border) found in ovarian, breast, and pancreatic PIE-MINs (Figs 4 d, 5d and 6d, respectively) are connected to hub genes. The complete list of Class II cancer genes and gene-gene connections in PIE-MINs in these cancer types is provided as Supplementary Data 6.

Further analysis of PIE-MIN genetic landscapes indicate the importance of Class II cancer genes in conveying oncogenic signals by connecting to differentially expressed hub genes. For instance, FBXW8, a gene with reported nonsense and splicing mutations, is a differentially expressed hub connecting a number of Class II genes in our ovarian cancer PIE-MIN (Fig. 4d). Another hub is ubiquitin C (UBC) with reported frameshift insertion and nonsense mutations. Both FBXW8 and UBC are key mediators in ubiquitin-mediated proteolysis. Of note, this process is also enriched in the ovarian cancer MIN (Fig. 2c) indicating the importance of protein homeostasis in ovarian cancer^41, 42^ and the role of Class II cancer genes (e.g. RNF7) in modulating this process in tumorigenesis. A number of long intergenic non-protein coding RNA genes (e.g. LINC00961, LINC00269) and uncharacterized RNA genes (e.g. LOC101927181, LOC284930) were found to be Class II genes. Of note, LINC00961 expression was recently shown to be enhanced in cisplatin-resistant high-grade serous ovarian cancer cells^43^ and overexpression of LINC00269 impaired migration and invasion capacities in a choriocarcinoma *in vitro* model^44^. These recent experimental findings indicate that altering Class II gene expression in cancers might alter cancerous behaviors, such as drug resistance and acquired invasion capability.

Our analyses also revealed differentially expressed hub genes, such as gene cassette CYP3A7-CYP3AP1, connect a substantial number of Class II genes in the breast cancer PIE-MIN (Fig. 5d). Class II genes associated with CYP3A7-CYP3AP1 cassette fall in the following major functional categories: (i) (DNA damage responses (FAM178A, RAD51, L3MBTL4), (ii) cytoskeleton remodeling (WDR90), (iii) transcriptional regulation (TNAP, ZC3H3, ZNF777, ZNF865, ADTRP, PDAP1, ZKSCAN5, ZBTB7A), (iv) transporters (SLC19A1), (v) protein processing and trafficking (GGA1), (vi) cellular energetic (ATAD3B), and (vii) RNA processing (FIP1L1). Among these, Class II cancer genes involved in transcriptional regulation are prevalent. For example, an allele of CYP3A7 is associated with poor outcomes in leukemia, breast and lung cancers^45^. Intriguingly, some of the Class II genes interacting with the CYP3A7-CYP3AP1 cassette, such as SLC19A1^46^, were reported to associate with clinical outcomes. Also, ATPase Family, AAA Domain Containing 3B (ATAD3B) was reported to significantly associate with poor survival in breast cancer patients^47^.

## Discussion

During the past two decades, machine learning methods have played important roles in biological discoveries^17^ including our group’s efforts to uncover the pharmacological properties of drugs^15, 16^. Machine learning approaches such as Support Vector Machine (SVM) and Artificial Neural Network (ANN) in general have the power to handle large-scale multi-dimensional data and extract hidden regularity or patterns that are relevant to a phenomenon from high-dimensional spaces^48, 49^. To harness the power of machine learning in combination with network and systems biology^50–52^, we designed the MALANI algorithm to identify key cancer genes in high-dimensional data space that would otherwise go undetected by conventional approaches. To our knowledge, this is the first study that utilizes both machine learning and network biology approaches to uncover “cryptic” genetic components, which we’ve termed Class II cancer genes, that coordinate functionality between Class I differentially expressed and frequently mutated cancer genes in cancer networks.

Our proof-of-principle study demonstrates MALANI can be employed to infer a set of gene pairs, whose dot product of gene expression values help discriminate between cancer vs. normal contexts. These gene pairs can then be agglomerated into a cancer-type specific network. Further characterization of MALANI-inferred networks (MINs) across various cancer types indicates they are indeed relevant to the processes involved in tumorigenesis. Cross-cancer comparison of MINs indicate context-dependent contributions from the tissues of cancer origin^53, 54^. These cancer type-specific components in MINs can be used as potential diagnostic markers and also as a means to improve understanding of how genes that modulate the activity of a tissue-specific network contribute to tumorigenesis^25^ and may be targeted for therapy development^55^.

Although gene-gene associations found in MALANI-derived cancer networks do not necessarily imply causality, our machine learning-based reverse engineered cancer networks provide key information regarding the existence of Class II cancer genes, which link to Class I cancer genes in order to complete oncogenic signaling in cancer networks. We next mapped reported mutational profiles from genome-wide studies for pancreatic, breast, and ovarian cancers onto respective MALANI-derived cancer networks to further characterize functional relevance of Class II cancer genes in cancer etiology. Our results indicate that gene pairs comprising a MIN play a role in coordinating the functionality of the cancer network and that non-differentially expressed and rarely mutated Class II genes indeed play an important role in conveying oncogenic signals in cancer networks. As such, perturbing Class II genes might impede functional coordination between hubs and frequently mutated genes, thus potentially rendering cancer cells more vulnerable.

The journey to understand the role of Class II cancer genes is still in its infancy. Although these gene candidates are blinded to methods that rely on mutational and gene expression information, we anticipate these Class II genes to exhibit differential activities in different functional aspects of cancer networks, namely, differential information flows as related to protein complexes^52^ and post-translational modifications, such as phosphorylation, to which further studies are warranted in the future. Machine learning-based reverse engineered networks, as illustrated in this work, can be extended to incorporate multi-omics data to reverse engineered cross-omics network in near future, where network-based context-specific multi-omics pathway enrichment methods such as KeyPathwayMiner 4.0^20, 56^ can be integrated to infer cross-omics key regulators in cancer etiology.

## Materials and Methods

### Datasets

We collected gene expression data corresponding to 9 cancer types from Gene Expression Omnibus (http://www.ncbi.nlm.nih.gov/geo/) for Affymetrix Human Genome U133 Plus 2.0 platform (Supplementary Table 1). A total of 6957 cancer and 1850 normal tissue arrays were collected (Supplementary Table 1 and Supplementary Data 1). In addition, a total of 288 liver cancer and 217 ovarian cancer arrays (Supplementary Data 3) were collected as independent datasets. The probe intensity from the microarray data was normalized as described in our previous published study^47^. In brief, intensity of probes were background corrected and probesets were summarized, but not normalized, using the R BioConductor rma function. The intensities of probesets mapped to the same gene were then averaged. Finally, each array was normalized by dividing the expression of each gene by the sum of all of the gene expression intensities per array, which resulted in overall gene expression of a given sample. Since this method normalizes each CEL file independently and produces the same results whether the CEL files are concurrently normalized, there is no batch effect. The R script can be obtained at https://github.com/pcahan1/CellNet/blob/master/cellnetr_preprocess.R.

### MALANI algorithm

The MALANI algorithm can be divided into three stages as shown in Fig. 1. The description below refers to computational steps for one cancer type; the same procedures were repeated for remaining cancer types in this study.

**Stage 1:** Construction of gene-wise models (Fig. 1a).

**Step 1.1:** Splitting of gene expression data matrix for 10-fold cross-validation. We defined a microarray data matrix after background correcting, summarizing, and normalizing.CEL files from all samples as ***M***_*G*×*S*_, where *G* represents the number of genes (rows in data matrix) and *S* represents samples (column in data matrix). The normalized data matrix for each cancer type was split into 10 portions, according to the total number of cancer and normal samples, respectively.

**Step 1.2:** Construction of gene-wise dot product matrices. During the first training fold, data from cancer and normal samples were split resulting in 9 portions that were used as training data and portion number 10 of split cancer and normal data were used as testing data. ***G*** is a set which contains all gene names in ***M***. We define *G* data matrices: ${M}_{(G-1)\times S}^{1},{M}_{(G-1)\times S}^{2},\ldots ,{M}_{(G-1)\times S}^{i},\ldots ,{M}_{(G-1)\times S}^{G}$, where rows in ${M}_{(G-1)\times S}^{i}$ are the dot product of the *i* ^*th*^ row with all other but that row in ***M***_*G*×*S*_. The *i* ^*th*^ gene *i* = 1 : *G* in ***G*** is represented by ${M}_{(G-1)\times S}^{i}$, and the idea is to find a set of selected genes, **Θ**, where **Θ** ⊂ **G** with a much smaller number of genes. For example, a gene-wise dot product matrix would be constructed for expression values of gene pairs for gene1, such as gene1-gene2, gene1-gene3, up to gene1-gene*G*. This gene-wise dot product matrix represents one SVM model for a particular gene. Dot product matrices were constructed for all genes (i.e. matrix rows) in the expression array and a total of *k* × *G* SVM models (*G* = 20075 genes, *k* = 10) corresponding to each gene were built.

**Step 1.3:** Model training and testing. Gene-wise dot product matrices generated from Step 1.2 for the first training fold were used to train SVM gene-wise models for their capability to classify cancer vs. normal samples. We used SVM function embedded in “e1071” R package with its default parameters. This function uses radial basis (exp(−gamma*|u − v|^2^)) as its default kernel function. The parameters are: SVM-type: C-Classification; SVM kernel: radial; cost = 1; gamma = 0.1666667. To train SVM gene-wise models in the second training fold, portion number 2 to 10 of split cancer and normal data were used as training data and portion number 1 of split cancer and normal data were used as testing to construct gene-wise dot product matrices as described in Step 1.2. The training procedure was repeated until 10^th^ training fold reached.

**Step 1.4:** Evaluation of gene-wise model performance. Gene-wise dot product models were assessed for their performance in classifying cancer vs. normal samples by computing average 10-fold cross-validation performance. Genes in ***G*** were further ranked based on average performances with the top 5% being categorized as selected gene set, **Θ**.

**Stage 2:** Construction of gene-pair models (Fig. 1b).

**Step 2.1:** Construction of gene-pair vector from **Θ** set.

Using split data from Step 1.1, vectors of gene pairs from a set of **Θ** genes obtained from Step 1.4 (denoted as red “g” in Fig. 1b) with remaining genes in the expression array denoted as black “g” in Fig. 1b) were constructed as follows: The expression data for preselected genes is denoted by ***P***_Θ**×***S*_, where **Θ** is the number of preselected genes equal to ≈5 × *G*/100 (Fig. 1a). Given ***g***_*i*_ ∈ **Θ** (*i* = 1 : Θ), we define two row data matrices, ${P}_{1}^{i},{P}_{2}^{i},\ldots ,{P}_{j}^{i},\ldots ,{P}_{G}^{i}$, where $${P}_{j}^{i}=[\begin{array}{c}{{\boldsymbol{P}}}_{{\rm{i}}\times S}\\ {{\boldsymbol{M}}}_{{\rm{j}}\times S}\end{array}]$$ and ***P***_i×*S*_ ≠ ***M***_j×*S*_. More specifically, there are (*G* − 1) gene-pair SVM models corresponding to each gene in **Θ** when it is paired with all others except itself.

**Step 2.2:** Model training and testing. Each gene-pair vector corresponding to a gene in set **Θ** was used to train SVM models during each training fold of a 10-fold cross-validation as described in Step 1.3. A total of *k* × (*G* − 1) SVM models (G = 20075 genes, *k* = 10) were generated in this stage.

**Step 2.3:** Evaluation of gene-pair model performance. The ability of gene-pair models to classify cancer vs. normal samples was computed using average 10-fold cross-validation. The top 10 best performing pairs were selected for each ***g*** ∈ **Θ**. Genes not in the **Θ** set (black “g” in Fig. 1b) that paired with genes in the **Θ** set (red “g” in Fig. 1b) and were among the top 10 in terms of best classification performance were deemed functionally associated with a given gene in set **Θ**. At the conclusion of this step, 10 × **Θ** pairs were kept.

**Stage 3:** Inferring a cancer network (Fig. 1c)

**Step 3.1:** Construction of finalized gene-pair dot product matrix. The dot products of gene expression for gene pairs obtained from Step 2.3 were used to construct a finalized single dot product matrix corresponding to each cancer type. The final data matrix was defined as ***Q***_R×*S*_, where *R* = 10 × Θ and each row represents one of the remaining pairs and is equal to the dot product of expression values of genes within that pair.

**Step 3.2:** Assessment of gene pair performance in classifying cancer samples. Five well-known feature selection (FS) methods, Information Gain (IG)^57^, Relief-F (RF)^58^, Summarized Uncertainty (SU)^59^, SVM-Recursive Feature Elimination (SVM-RFE)^60^ as well as a simple method called Individual Accuracy (IA)^61^, are used to select best feature pairs in ***Q*** from step 3.1. Each FS method is applied independently and ranks the features (pairs) based on the specific method. These FS methods were used to assess performance of each gene pair dot product in classifying cancer vs. normal samples. Gene pairs shown to contribute to a gain (i.e. improved) in classification performance in a majority of feature selection methods, at least three of the five (Supplementary Data 3), were deemed cancer-related genes (Fig. 1c). Further information on evaluation of feature selection methods can be found in our recent work^61^.

**Step 3.3:** Construction of MALANI-Inferred Network (MIN) for a cancer. Gene pairs selected in Step 3.2 were then agglomerated to reconstruct an inferred cancer network.

### Computational complexity of MALANI algorithm

MALANI uses about $k\times G\times (1+\frac{G-1}{20})$ SVM models before constructing ***Q*** and applying FS methods to rank the gene pairs where k represents the k-fold cross validation scheme. Furthermore, the number of samples in each microarray dataset also affects the complexity of computations in constructing ***Q***. We used Linux-based High Performance Computing (HPC) environment at Mayo Clinic, Research Computing Services (RCS), 64 cores Intel(R) Xeon(R) CPU E5-4650 v2 @ 2.40 GHz, 256 GB of RAM to build ***Q***. Average running time for each cancer data set in our study was ~60 hours.

### Model evaluation

Due to the computational complexity of the MALANI algorithm as mentioned above, we used a model built from pancreatic cancer data for model evaluation. We used permutation tests and multiple testing for model evaluation. For permutation tests, sample labels were randomly swapped and a SVM classifier was used for gene-wise models at Stage 1. 10-fold cross-validation was used for final model evaluation. We ran the simulations 20 times with different seeds to permutate sample labels. Monte-Carlo permutation test was used to estimate the P-value. Confidence interval (CI) around the observed P-value was estimated as:

$$\begin{array}{rcl}95 \% {\rm{CI}} & = & {\rm{\alpha }}\pm 1.96\ast \sqrt{\frac{{\rm{\alpha }}(1-{\rm{\alpha }})}{M}}\\ Observed\,P-value\,or\,{\rm{\alpha }} & = & \frac{\{\#|{M}^{Permutation}|\ge |{M}^{Observation}|\}}{\#\,{\rm{of}}\,{\rm{Permutations}}}\end{array}$$

where α is the observed P-value and M is the number of permutations. Our results (Supplementary Figure 2) show that there are indeed significant differences between selected genes with true labels compared with permutated synthetic data and the estimated P-value and 95% CI are close to zero.

Pancreatic cancer was used as a study model for multiple testing. Raw p-values were computed based on 570 gene pairs (344 unique genes) selected at Stage 3 and multiple testing was performed to find out the percentage of statistically significant genes in this gene list. “genefilter” in R package was used to compute the parametric p-values for all 344 unique genes selected at Stage 3. Next, we adjusted the computed p-values for multiple testing. Bonferroni and Benjamini-Hochberg (BH), which are respectively used to control family wise error (probability of observing one false positive) and to control false discovery rate (FDR) were used for multiple testing correction. Results of multiple testing are provided in Supplementary Figure 3. Considering a total of 20075 genes in our transcriptomic data set, the significant percentages are 64.2%, 61% and 27.3% for raw p-values, BH and Bonferroni tests, respectively while these percentages are 86.6%, 86% and 67.7% in MALANI’s list. This indicates that MALANI can condense large number of genes (20075 in this work) to those statistically significant genes in the cancer model.

### Testing the feasibility of the MALANI algorithm with different machine learning methods and feature selection approaches

To illustrate the feasibility of MALANI using machine learning methods other than SVM, we repeated the MALANI algorithm using Random Forest (RF) and Logistic Regression (LR) classifiers on the pancreatic dataset and compared genes selected by 3 classifiers (SVM, LR, RF) at Stage 1 for genes in set **Θ** and genes selected at Stage 3 for the finalized gene-pair matrix. Default parameters of “randomForest” of R package and “glm” function in R “stats” package were used to implement Random Forest (RF) and generalized liner models (LR), respectively. Using Fisher’s exact test, our results show strong statistical consistency across these 3 classifiers (Supplementary Figure 4).

To examine the robustness of selected features at Stage 3 and to compare feature genes selected via a vote by majority strategy using 5 feature selection methods vs. an ensemble of feature selection methods (EFS)^62^, we then applied R package of *EFS v1*.*0*.*1*, on pancreatic cancer data. Our results indicate 275 features are commonly selected by both strategies, whereas 236 features are unique to both strategies, respectively. Fisher’s exact test provides a statistical significance (p-value = 4.940656e-324) for features selected by both approaches, indicating that selected features are robust between vote by majority approach and ensemble of feature selection method.

### Pathway enrichment

Pathway analyses were performed using a web-based engine at WebGestalt (**WEB**-based **GE**ne **S**e**T A**na**L**ysis **T**oolkit: at http://bioinfo.vanderbilt.edu/webgestalt/) using gene symbols mapped to the human genome. Canonical pathways from a KEGG database were queried. All genes in the human genome were used as a reference set for enrichment analysis. Over-representation of genes present in a given pathway was assessed using a hypergeometric test followed by Bonferroni correction for multiple tests. Enrichment was computed as follows: Suppose we have n genes in an interested gene set A and m genes in the reference gene set B. And suppose that there are k genes in A and j genes in B that are in a given category C (e.g. a KEGG pathway). Based on the reference gene set, the expected value of k would be k-expected = (n/m)* j. If k exceeds the expected value, category C is said to be enriched, with a ratio of enrichment (r) given by r = k/k-expected. For enrichments of genes residing in MINs, the top 10 enriched pathways with at least 3 genes present from the submitted gene list were deemed significant. For enrichment of genes residing in PIE-MINs, pathways with adjusted p-value < 0.05 with at least 5 genes present from the submitted list were considered statistically significant.

### Search for shortest protein-protein interaction paths encapsulated by an MIN gene pair

All connecting paths to a gene pair can be relevant to cancer activities, however, due to computational complexity and cost of computation, it is commonly assumed that the shortest path provides minimal cost and maximal desired activity^63, 64^. From a biological standpoint, we reason that the shortest protein-protein interaction paths connecting gene pairs are the most biologically relevant based on a random walk algorithm applied in previous publications^65, 66^. Here, R package *igraph ver 1*.*0*.*1* was used to find the shortest path between MIN genes. Since no weight constraints were assigned to the edges in the PPI network, Dijkstra’s algorithm^67^ was used to calculate the shortest paths. For example DDX39 A and PRM1 were selected in the feature selection step by all methods, except SVM-RFE, as an important pair of genes for ovarian cancer. Figure 3a provides an illustration of the shortest Protein-Protein interaction (PPI) paths encapsulated by the DDX39A-PRM1 gene pair. Given this small PPI network, there are 1,145,788 possible undirected paths to connect the pair of interest. The length of the paths vary from 9 to 24 proteins. However, there is only 1 path the length of 9 proteins – U2AF2-PTBP1-CASP8-FOXO3-SP1-MYCN-KDM3A – as reported by Dijkstra’s algorithm as the shortest path. The same procedure was applied for all gene pairs in MINs, resulting in networks that we defined as Protein-Protein Interactions Encapsulated by MINs (PIE-MINs).

### Construction of PIE-MIN genetic landscape

As explained above, a PIE-MIN gene list contains all **G**_**i**_-**G**_**j**_ gene pairs within a MIN and genes in the shortest possible path between those pairs within the overall PPI network. Reported mutated genes from genome-wide studies (Supplementary Data 4) were used to map to PIE-MINs of ovarian, breast, and pancreatic cancers. For example, of the 622 gene pairs present in the ovarian cancer network, 435 of the genes are unique with 526 unique genes within the shortest paths between those pairs. As such, there are 961 genes comprising the PIE-MIN list, of which 400 genes are known to have the missense mutation (Fig. 4a). Each one of those 400 genes has its own neighbor genes resulting in a total of 51350 direct neighbor genes (Fig. 4b). Also, each of PIE-MIN genes (961 genes) can be found in 0 to 10 mutation’s gene list, i.e. except Amplified and Intronics, TP53 is a common gene in the remaining ovarian mutation types (Fig. 4c). Within the 435 unique MIN genes there are 165 genes which are either Class II genes or are up/down regulated; in this case, there are 213 total genes in the PIE-MIN list. We further used the 106 genes which have a p-value greater than the 3rd quantile of all p-values in the 213-gene list to construct the network (Fig. 4d). A similar procedure was applied to construct PIE-MIN genetic landscapes for breast and pancreatic cancers (Figs 5 and 6). The mapped mutated genes in PIE-MINs of ovarian, breast, and pancreatic cancers are provided in Supplementary Data 5. Class II cancer genes detected within the shortest paths connecting gene pairs in MINs of ovarian, breast, and pancreatic cancers are indicated in Figs 4 d, 5d and 6d, respectively. Fisher’s Exact Test was then used to determine whether hubs in PIE-MINs significantly connect to Class II cancer genes (Supplementary Table 3) using 11521 proteins present in the KEGG databse as comparison background.

## Source Code

The MALANI source code is freely available at GitHub (https://github.com/HuLiLab/malani) and an online website portal (www.malani.hulilab.org/) was built to include the comprehensive step-by-step tutorial to guide users to download, install the source code, run the software locally, and better use the software to interpret MALANI results. An online forum group is also available under Google Groups (https://groups.google.com/forum/#!forum/malani-grp) to report bugs, troubleshot problems, or discuss the software online.

## Electronic supplementary material

Supplementary Information

Supplementary Dataset 1

Supplementary Dataset 2

Supplementary Dataset 3

Supplementary Dataset 4

Supplementary Dataset 5

Supplementary Dataset 6

Supplementary Tables
