# Supplementary material for: Machine Learning-Assisted Network Inference Approach to Identify a New Class of Genes that Coordinate the Functionality of Cancer Networks
Source: Sci Rep. 2017 Aug 1;7:6993. doi: 10.1038/s41598-017-07481-5 (PMC5539301; doi:10.1038/s41598-017-07481-5)

Machine Learning-Assisted Network Inference Approach to Identify a New Class of Genes that Coordinate the Functionality of Cancer Networks

Mehrab Ghanat Bari[1,3], Choong Yong Ung[1,3], Cheng Zhang[1], Shizhen Zhu[2] and Hu Li[1,*]

[1]Department of Molecular Pharmacology and Experimental Therapeutics, [2]Department of Biochemistry and Molecular Biology, Mayo Clinic College of Medicine, Rochester, MN 55905, USA.

[3]These authors contributed equally to this work.

*Corresponding author:

Hu Li, Ph.D.

Department of Molecular Pharmacology

and Experimental Therapeutics

Mayo Clinic College of Medicine

Gonda Building, 19-408

200 First Street SW

Rochester, MN 55905

Office: 1-507-293-1182

Fax: 1- 507-284-4455

E-mail: li.hu@mayo.edu

SUPPLEMENTARY LEGEND

Supplementary Figure 1. **MALANI-inferred networks (MINs) for 9 cancer types**. Top 25% of MIN genes with –log2(p-value) were selected to build the network. In other words, the node sizes are correlated with –log2 of p-values. Gene pairs selected by 5, 4, and 3 feature selection methods are indicated in red, green, and grey edges, respectively. Nodes size changes based on –log2 of p-value scores, and color of nodes alters by fold change of its corresponding gene, where fold change in the ranges $(0,0.5], (0.5,0.66], (0.66,1.5), [1.5,2), [2,\text{inf}\ )$ are shown in cryptic blue, light blue, gray, purple and red.

Supplementary Figure 2. **Permutation test on pancreatic cancer model**. Boxplot of top 5% of selected genes that contribute to classification performance in Stage 1 with true and permutated labels.

Supplementary Figure 3. **Multiple testing on pancreatic cancer model**. (**a**) Raw p-avlues. (**b**) Benjamini –Hochberg corrected p-values. (**c**) Bonferroni corrected p-values. (**d**) The percentage of statistically significant genes considering raw p-avalue, Bonferoni and BH-corrected significant level of 0.05%.

Supplementary Figure 4. Performance of MALANI algorithm on different classifiers. (**a**) Accuracy range of final selected pairs. (**b**) MALANI's run time with different classifiers.

Supplementary Table 1. **Cancer types and number of expression arrays**.

Supplementary Table 2. **Statistics of differentially and non-differentially expressed genes and cryptic gene candidates in MINs across diverse cancer types**.

Supplementary Table 3. Fisher's Exact Test for ovarian, breast, and pancreatic cancer PIE-MINs for hub genes (> 5 connections) that connect to Class II genes.

Supplementary Data 1. Array IDs of 9 cancer types and their corresponding normal control arrays used in this study. Information of replicate samples is provided in "Replicate samples" sheet.

Supplementary Data 2. Complete gene pairs selected by at least 3 feature selection methods that constitute the MALANI-Inferred Networks (MINs).

Supplementary Data 3. Independent data test sets.

Supplementary Data 4. Reported gene mutations from genome-wide studies for ovarian, breast, and pancreatic cancers.

Supplementary Data 5. Reported genome-wide mutated cancer genes mapped to PIE-MINs of ovarian, breast, and pancreatic cancers.

Supplementary Data 6. Lists of Class II genes and gene-gene connections in PIE-MINs of ovarian, breast, and pancreatic cancers.

Supplementary Discussion: On the effect of tissue-specific contexts to the performance of MALANI-derived models.

Supplementary Discussion: On the effect of tissue-specific contexts to the performance of MALANI-derived models

The etiology of cancer had been reported to associate to tissue-specific context although a number of oncogenes such as RAS and tumor suppressor genes such as TP53 are reported to involve in tumorigenesis in broad cancer types. To understand whether MALANI captures tissue-specific signals that are potentially to play a role in tumorigenesis, we test MALANI with the following two strategies: (i) use of cross-tissue type normalized samples instead of tissue-dependent normal samples to train and classify cancers derived from their respective tissue types; and (ii) use top most frequent genes selected by MALANI from a respective tissue type, and test their classification performance across all 9 cancer types.

In strategy (i), we used normal samples from pancreas, prostate, and liver as a case study to survey the effect of using normal samples normalized across all tissues (regardless of tissue types) in respect to pancreatic, prostate, and liver cancers. We concatenated 480 normal samples which belong to Pancreatic (n=81), Prostate (n=90) and Liver (n=309). Then we normalized

them and substituted with original normal samples in mentioned cancer type. Finally we applied MALANI on each cancer type separately and compared the selected genes with genes were selected using original normal samples.

Supplementary Discussion Figure 1 | Data reading for pancreatic, prostate, and liver normal vs. cancer samples (a) before and (b) after normalization.

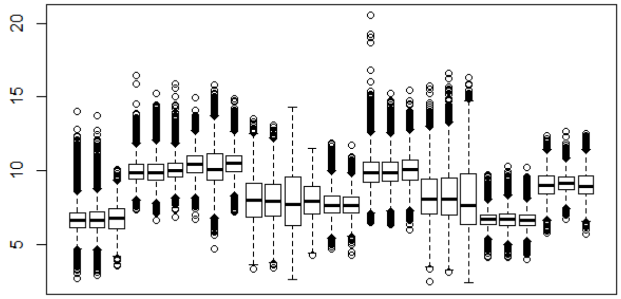

a) Before normalization

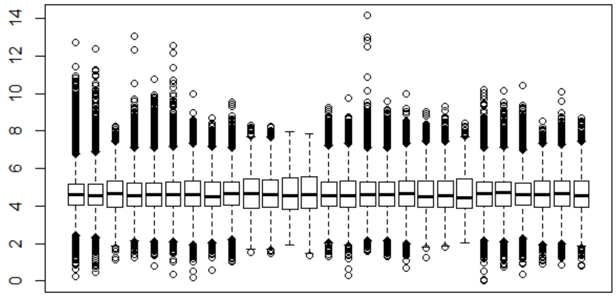

b) After normalization

Let's define:

List1: 1003 preselected genes in MALANI's first step when original samples (i.e. tissue-specific) were used

List2: 1003 preselected genes in MALANI's first step when 480 normal samples (i.e. regardless of tissue types) were used.

As shown in Supplementary Discussion Table 1, there are general drop in classification accuracy using cross-tissue normalized normal samples for classifying pancreatic, prostate, and liver cancers, suggesting tissue-specific contexts are indeed relevant in etiology specific to different cancer types. This illustrates MALANI is capable to indicate the importance of the contribution of tissue-specific contexts when tissue type of cancer is considered in the model building procedure.

Supplementary Discussion Table 1 | Performance of classification of MALANI models when tissue-specific normal samples (List1) and cross-tissue (pancreatic, prostate, and liver) normalized normal samples (List2) were used to classify samples from pancreatic, prostate, and liver cancers, respectively.

	**Prostate**		**Pancreatic**		**Liver**	
**# of features**	**List1**	**List2**	**List1**	**List2**	**List1**	**List2**
**2**	83.8	72.8	90.1	83.8	64.8	62.3
**3**	86.1	77.8	96.6	89.7	73.1	63.6
**4**	90	80.6	97.3	90.4	72.4	64.0
**5**	91.5	81.1	98.1	92.6	88.6	63.8
**6**	91.8	82.1	98.9	93.9	97.3	79.0
**7**	93.3	85.8	99.2	94.8	99.1	79.6
**8**	95.4	87.9	100	95.7	99.7	84.6
**9**	94.3	90.9	99.6	95.9	99.5	85.1
**10**	96.4	90.5	99.6	95.9	99.8	86.3
**11**	98.5	91.3	99.6	96.8	99.7	86.6

In strategy (ii), we reason that if different types of cancers differ in genes that are important, this can be evaluated by their capability in classification, with highest classification accuracy is expected for genes derived from cancer of same tissue types. Here, we used top 5 genes with highest frequencies for their occurrences in MALANI-selected gene pairs to test this assumption across all 9 cancer types. Supplementary Discussion Table 2 shows the results using top 5 most frequent genes found in MALANI-derive networks across pairs of cancer types. Some of these top 5 genes can be overlapped between cancer types. In general, genes from same cancer type yield highest classification accuracy (reds) for pairs of same cancer type. Interestingly, we found general drop of classification accuracy for pairs of different cancer types when we used top 5 most frequent genes that are unique to each cancer type (Supplementary Discussion Table 3). This indicates cancer type-specific genes do differ in genes that are important.

Supplementary Discussion Table 2 | Classification accuracies using top 5 genes with highest frequencies for their occurance in MALANI-selected gene pairs. Red: Highest classification accuracy; bold: classification on same cancer type.

	**pancreas**	**breast**	**colon**	**kidney**	**liver**	**lung**	**ovary**	**prostate**	**skin**
**pancreas**	**99.6**	98.2	91.5	94.9	89.1	98.2	98.3	92.9	97.4
**breast**	99.6	**99.0**	97.4	92.9	84.7	97.1	99.0	91.3	98.5
**colon**	93.4	83.5	**99.9**	93.7	96.8	99.7	97.1	90.7	99.3
**kidney**	91.1	83.8	99.0	**99.6**	91.3	97.0	97.3	95.6	97.3
**liver**	87.6	89.7	96.5	96.3	**99.0**	98.9	94.9	95.5	97.3
**lung**	98.4	93.3	93.8	91.3	91.2	**99.8**	98.5	90.0	96.9
**ovary**	95.7	96.1	98.1	96.2	81.1	97.4	**99.7**	90.8	99.2
**prostate**	94.2	83.8	96.4	95.5	87.6	97.8	96.9	**100**	99.5
**skin**	97.6	77.9	99.7	97.5	88.3	99.0	94.7	99.4	**100**

Supplementary Discussion Table 3 | Classification accuracies using top 5 genes with highest frequencies for their occurance in MALANI-selected gene pairs which are unique to each cancer type. Red: Highest classification accuracy; bold: classification on same cancer type.

	**pancreas**	**breast**	**colon**	**kidney**	**liver**	**lung**	**ovary**	**prostate**	**skin**
**pancreas**	**99.2**	84.8	91.9	89.7	84.4	92.0	95.3	91.3	93.9
**breast**	83.0	**97.2**	92.1	88.5	84.5	87.8	94.6	90.5	95.9
**colon**	82.3	76.4	**99.8**	88.5	87.7	95.5	92.8	93.2	97.5
**kidney**	81.9	75.6	97.5	**99.6**	86.1	91.4	94.6	95.8	96.1
**liver**	91.5	86.4	97.6	93.4	**98.5**	96.4	95.3	92.6	96.6
**lung**	91.9	76.3	97.3	81.2	87.9	**99.6**	95.8	91.9	96.3
**ovary**	83.7	87.9	94.8	89.8	78.5	95.4	**99.8**	90.7	95.0
**prostate**	72.2	77.9	97.3	95.7	89.6	88.4	93.0	**98.4**	93.7
**skin**	80.6	79.7	98.7	93.9	87.1	97.1	94.9	90.2	**100**

Supplementary Figure 1

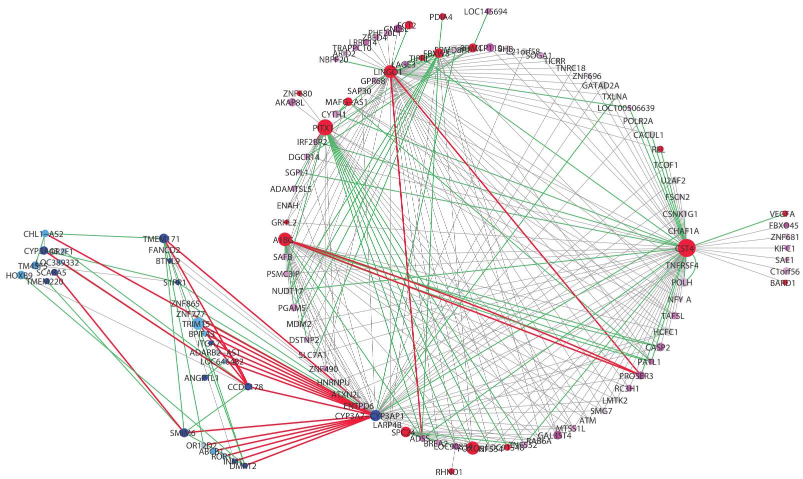

Breast

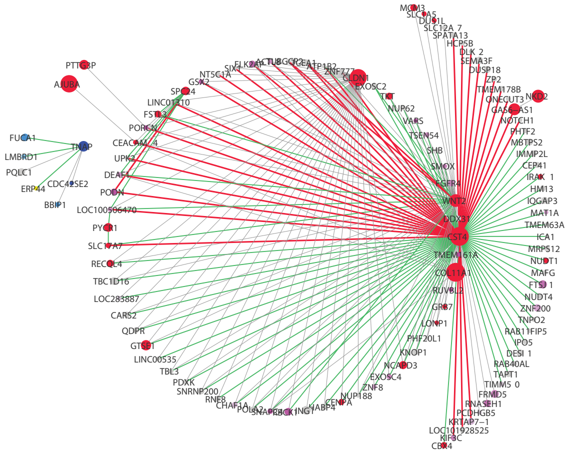

Colon

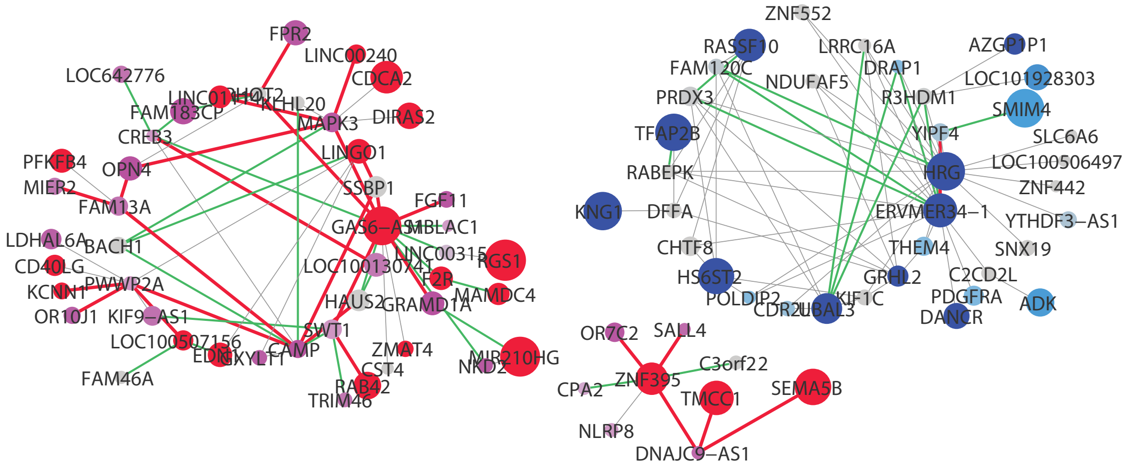

Kidney

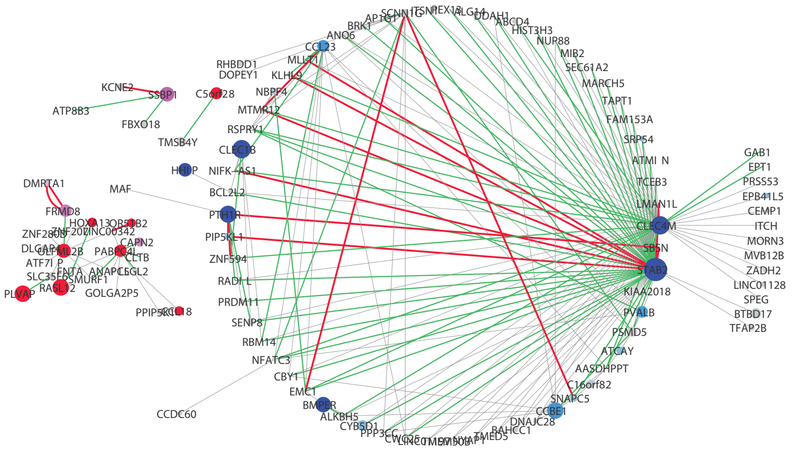

Liver

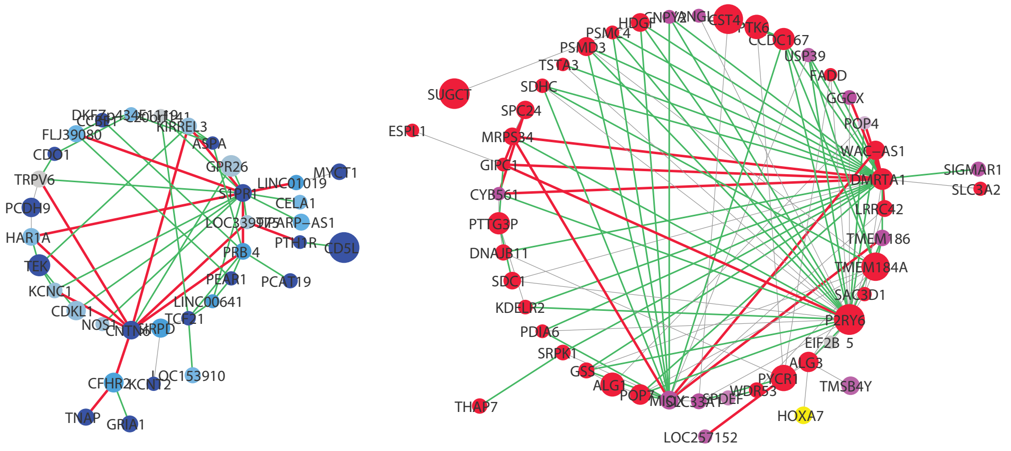

Lung

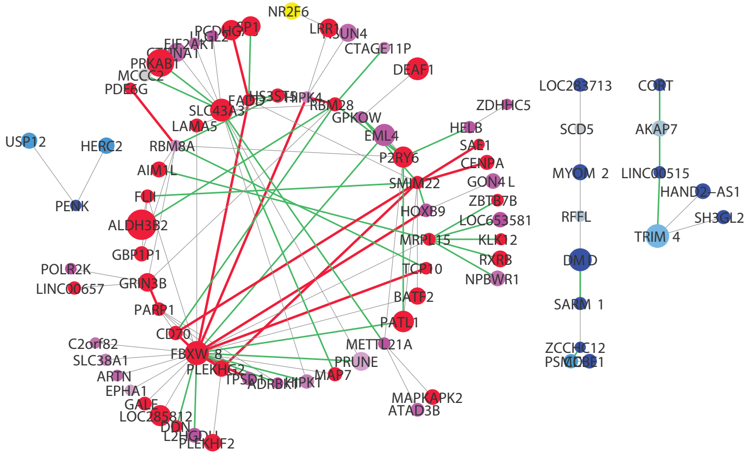

Ovary

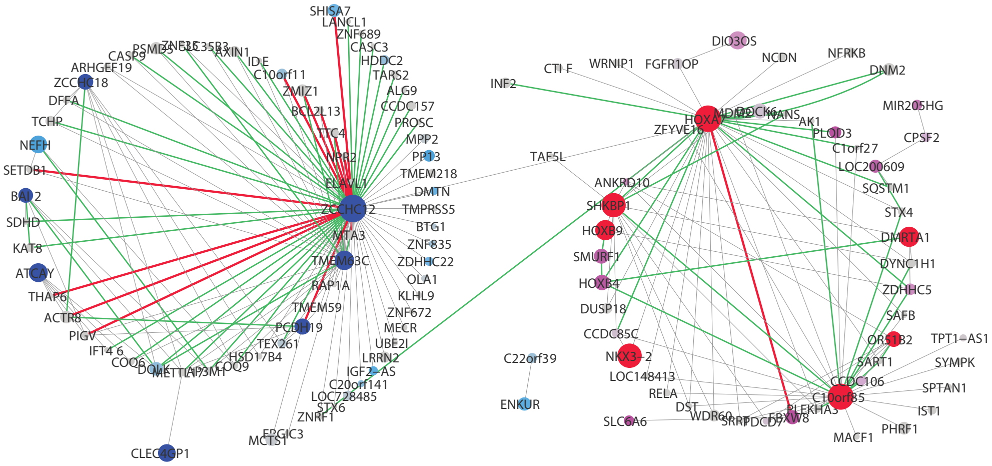

Pancreatic

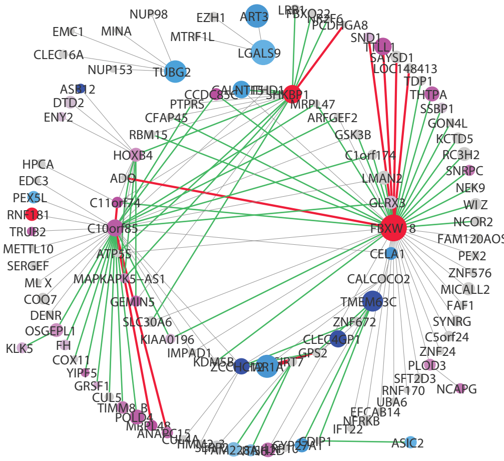

Prostate

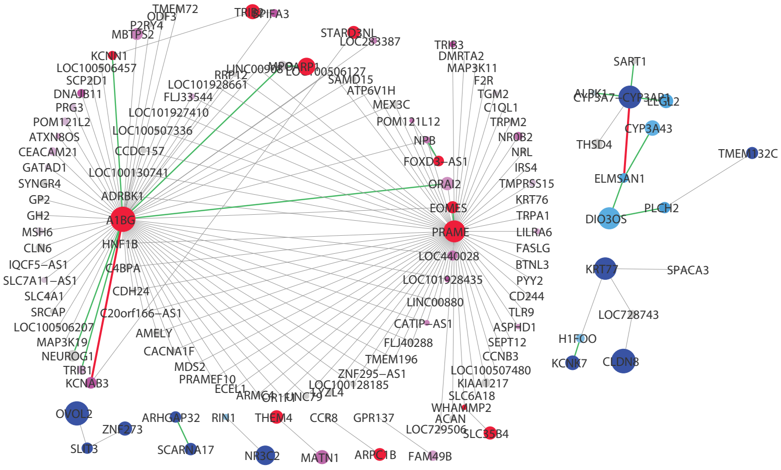

Skin

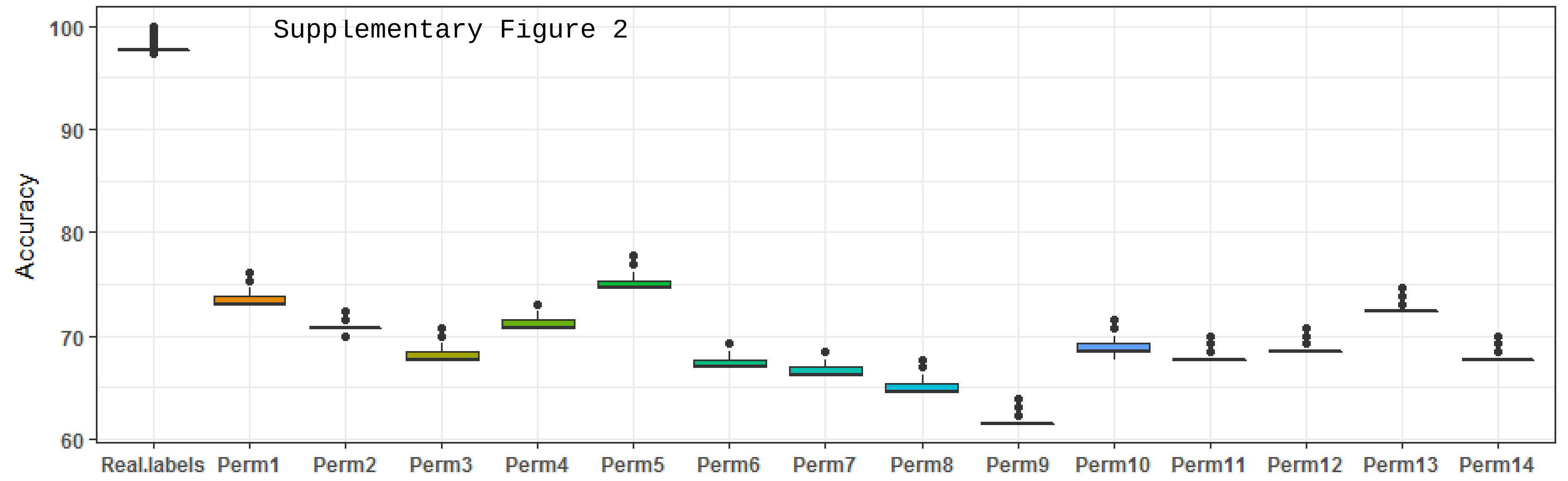
Supplementary Figure 2
Accuracy
100
90
80
70
60
Real.labels
Perm1
Perm2
Perm3
Perm4
Perm5
Perm6
Perm7
Perm8
Perm9
Perm10
Perm11
Perm12
Perm13
Perm14

Supplementary Figure 3

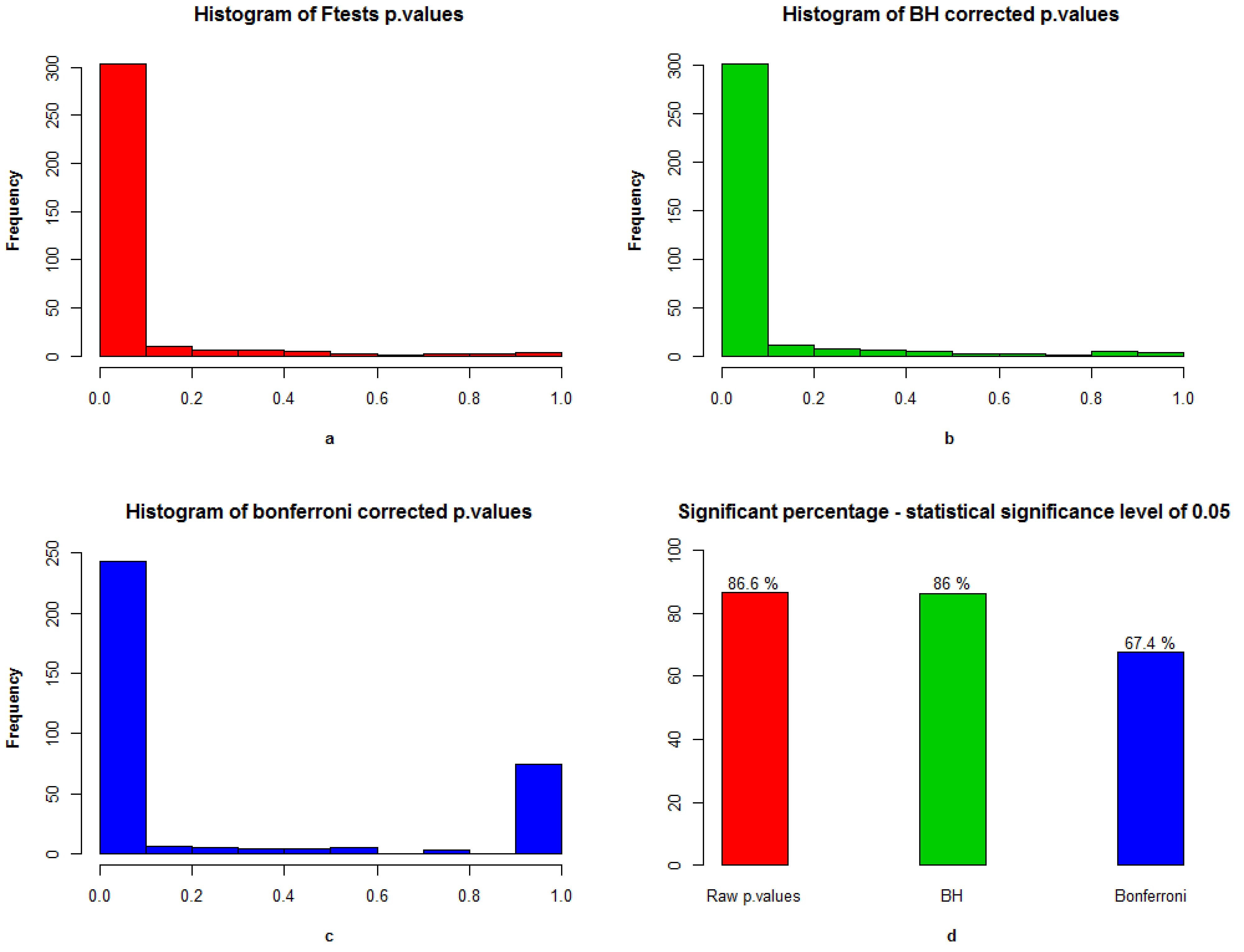

Supplementary Figure 4

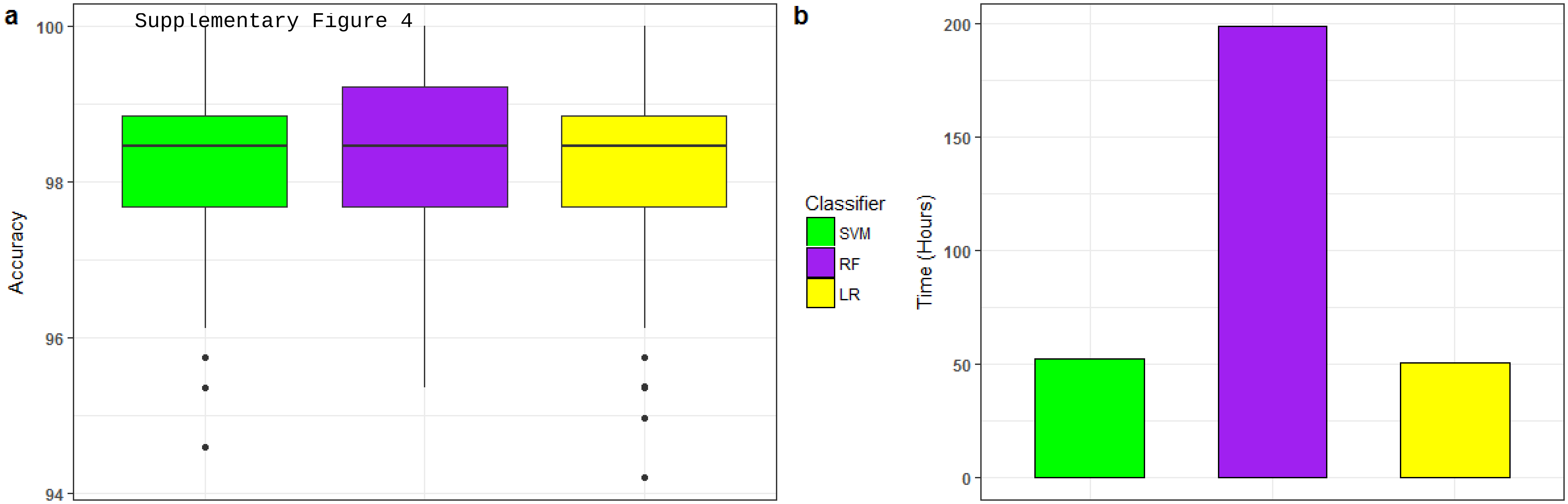

Supplement: Supplementary file 1 — Supplementary Information [file 41598_2017_7481_MOESM1_ESM.pdf]
